# Aluminium phosphide (Al_12_P_12_) nanocage as a potential sensor for volatile organic compounds: A DFT study†

**DOI:** 10.1039/d4ra01828a

**Published:** 2024-04-29

**Authors:** Mahmoud A. A. Ibrahim, Manar H. A. Hamad, Nayra A. M. Moussa, Omar H. Abd-Elkader, Shaban R. M. Sayed, Muhammad Naeem Ahmed, Ahmed M. Awad, Tamer Shoeib

**Affiliations:** a Computational Chemistry Laboratory, Chemistry Department, Faculty of Science, Minia University Minia 61519 Egypt m.ibrahim@compchem.net; b School of Health Sciences, University of KwaZulu-Natal Westville Campus Durban 4000 South Africa; c Department of Physics and Astronomy, College of Science, King Saud University P.O. Box 2455 Riyadh 11451 Saudi Arabia; d Department of Botany and Microbiology, College of Science, King Saud University P.O. Box 2455 Riyadh 11451 Saudi Arabia; e Department of Chemistry, The University of Azad Jammu and Kashmir Muzaffarabad 13100 Pakistan; f Department of Chemistry, California State University Channel Islands Camarillo California 93012 USA; g Department of Chemistry, The American University in Cairo New Cairo 11835 Egypt t.shoeib@aucegypt.edu

## Abstract

The efficacy of aluminium phosphide (Al_12_P_12_) nanocage toward sensing methanol (MeOH) and ethanol (EtOH) volatile organic compounds (VOCs) was herein thoroughly elucidated utilizing various density functional theory (DFT) computations. In this perspective, MeOH⋯ and EtOH⋯Al_12_P_12_ complexes were investigated within all plausible configurations. According to the energetic features, the EtOH⋯Al_12_P_12_ complexes exhibited larger negative values of adsorption and interaction energies with values up to −27.23 and −32.84 kcal mol^−1^, respectively, in comparison to the MeOH⋯Al_12_P_12_ complexes. Based on the symmetry-adapted perturbation theory (SAPT) results, the electrostatic forces were pinpointed as the predominant component beyond the adsorption process within the preferable MeOH⋯ and EtOH⋯Al_12_P_12_ complexes. The findings of the noncovalent interaction (NCI) index and quantum theory of atoms in molecules (QTAIM) outlined the closed-shell nature of the interactions within the studied complexes. Substantial variations were found in the molecular orbitals distribution patterns of MeOH/EtOH molecules and Al_12_P_12_ nanocage, outlining the occurrence of the adsorption process within the complexes under investigation. Thermodynamic parameters were denoted with negative values, demonstrating the spontaneous exothermic nature of the most favorable complexes. New energy states were observed within the extracted density of states plots, confirming the impact of adsorbing MeOH and EtOH molecules on the electronic properties of the Al_12_P_12_ nanocage. The appearance of additional peaks in Infrared Radiation (IR) and Raman spectra revealed the apparent effect of the adsorption process on the features of the utilized sensor. The emerging results declared the potential uses of Al_12_P_12_ nanocage as a promising candidate for sensing VOCs, particularly MeOH and EtOH.

## Abstract 2

The potentiality of the aluminium phosphide (Al_12_P_12_) nanocage as a prospective sensor material towards methanol (MeOH) and ethanol (EtOH) molecules was investigated using density functional theory computations.

## Graphical abstract

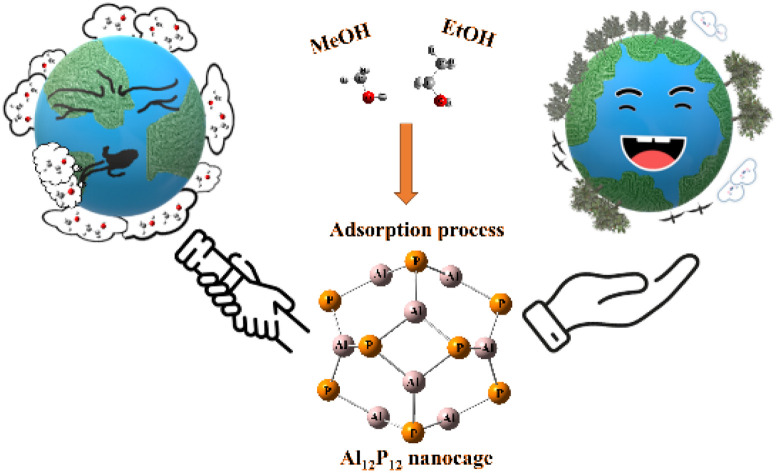

## 1. Introduction

In the contemporary world, scientists have focused significantly on developing sustainable nanomaterials for various applications, including energy, environment, and drug delivery. Indeed, the continued life of our planet depends significantly on advancements in sophisticated materials science. Several nano-based structures have been accordingly developed and were widely investigated, comprising fullerenes,^1^ nanotubes,^2^ and nanocones.^3^

More recently, the utilization of phosphide nanocages has attracted the attention of scientists.^4–6^ It is worth mentioning that inorganic aluminium phosphide (Al_12_P_12_) nanocage was earlier distinguished by its distinctive chemical features, including a high energy gap and low electron attraction.^7–9^ The potential applications of Al_12_P_12_ in non-linear optics,^10^ drug carriers,^11,12^ and sensors^13,14^ have garnered tremendous attention. As an appropriate sensing material, the adsorption of cyanogen chloride and hydrogen cyanide toxic gases on the surface of Al_12_P_12_ was investigated.^15^ Moreover, the sensitivity of the Al_12_P_12_ nanocarrier toward detecting phosgene gas was revealed.^16^

The increase in energy consumption and industrial activities made monitoring air pollution an essential priority for several countries and organizations.^17–23^ As a point of departure, volatile organic compounds (VOCs) are categorized as organic compounds with low water solubility, low boiling points, and high vapor pressure.^24,25^ Detailedly, VOCs are considered hazardous air pollutants and are among the most frequent air pollutants released by industrial chemical processes, standard household products, and construction materials.^26–28^ The natural environment and human health are significantly threatened by VOCs, which act as precursors to ozone and photochemical smog.^29–31^ VOCs are the main contributor to the greenhouse effect and also have the possibility to damage the human nervous and circulatory systems.^32,33^ Considering the risks of VOCs exposure, these compounds need to be eliminated from the environment. Several VOCs purification techniques have been developed, including adsorption,^34,35^ biodegradation,^36^ and membrane separation.^37^ Numerous alcohols, including methanol (MeOH), ethanol (EtOH), isopropanol, ethylene glycol, *etc.*,^38^ are commonly found as VOCs in various indoor air conditions. The adsorption amplitude of the MoSe_2_ nanosheet and carbon nanopores toward adsorbing the MeOH and EtOH was earlier divulged.^39,40^ Notwithstanding the promising properties of aluminium-bearing nanocages, no solid investigation revealed their efficiency in detecting the MeOH and EtOH molecules.

In this regard, the principal purpose of this study was to elucidate the potentiality of Al_12_P_12_ toward sensing the MeOH and EtOH molecules within all plausible configurations of the MeOH⋯ and EtOH⋯Al_12_P_12_ complexes. Geometrical optimization and frequency computations were executed for all the investigated systems, accompanied by adsorption and interaction energy calculations. To shed light on the physical forces dominating the adsorption process, the SAPT method was employed. Subsequently, the thermodynamic features, global indices of reactivity, and electronic parameters were assessed. This study will provide significant principles for the design and enhancement of Al_12_P_12_ nanocage applications in detecting toxic molecules, particularly for VOCs.

## 2. Results and discussion

### 2.1. ESP analysis

**Fig. 1:**
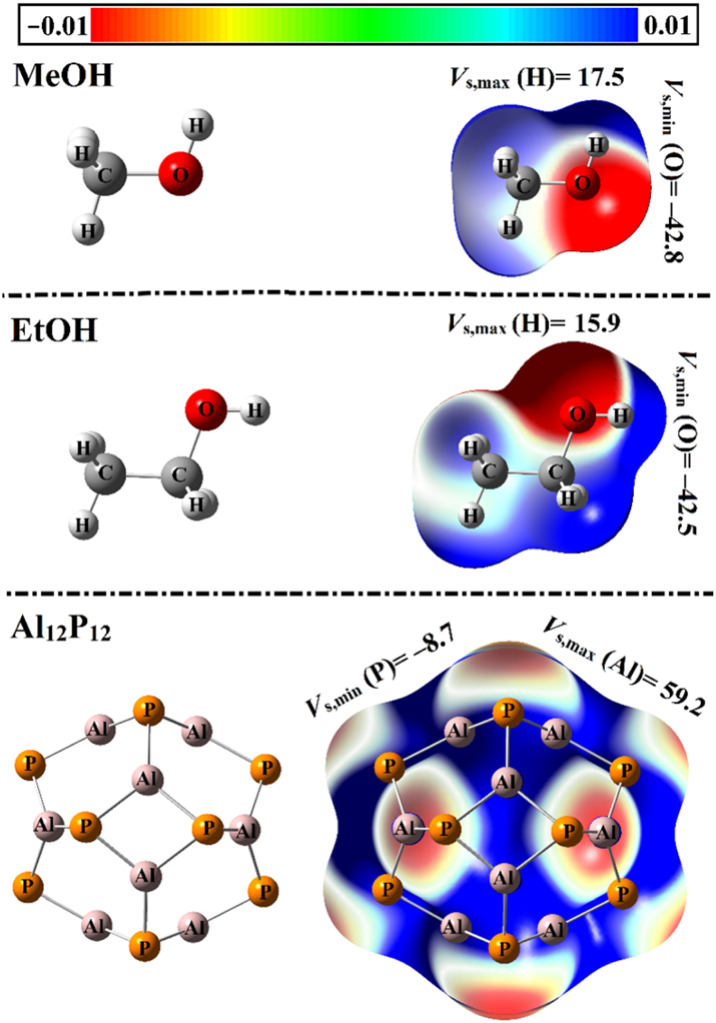
Optimized structures of VOCs and Al_12_P_12_ nanocage combined with the MEP maps and *V*_s,min_/*V*_s,max_ values (in kcal mol^−1^).

Molecular electrostatic potential (MEP) maps were portrayed for the optimized structures to clarify the electrophile and nucleophile sites, as recommended in literature.^41^ Different colors were utilized to depict the difference in ESP at the molecular surface. In the colored scale, the red/orange/yellow, green, and blue colors-refer to electron-rich, neutral, and electron-deficient sites, respectively. A quantitative insight was subsequently provided by computing the surface electrostatic potential extrema values (*V*_s,min_/*V*_s,max_) over the surface of the optimized monomers. Fig. 1 illustrates the MEP maps and *V*_s,min_/*V*_s,max_ values.

As displayed in Fig. 1, the pure Al_12_P_12_ nanocage exhibits *T*_h_ symmetry and is composed of eight hexagonal rings in combination with six tetragonal rings.^42^ At first glance, two different bonds in the Al_12_P_12_ nanocage were denoted. The first Al⋯P bond is shared by tetragonal and hexagonal rings with an average bond length of 2.28 Å. Meanwhile, the other Al⋯P bond is located between two hexagonal rings with a bond distance of 2.33 Å. The MEP plots of the MeOH and EtOH clarified the nucleophilic site at the region enclosing the O atoms. Such pictorial outcomes were ensured by the existence of negative *V*_s,min_ values of −42.8 and −42.5 kcal mol^−1^ over the surface of the MeOH and EtOH, respectively. At the same time, the electrophilic sites were observed *via* the existence of blue-colored regions around C and H atoms in the MeOH and EtOH molecules. On the surface of the Al_12_P_12_ nanocage, the red-colored nucleophilic and blue-colored electrophilic regions were around P and Al atoms with *V*_s,min_ and *V*_s,max_ values of −8.7 and 59.2 kcal mol^−1^, respectively.

### 2.2. Adsorption features

**Fig. 2:**
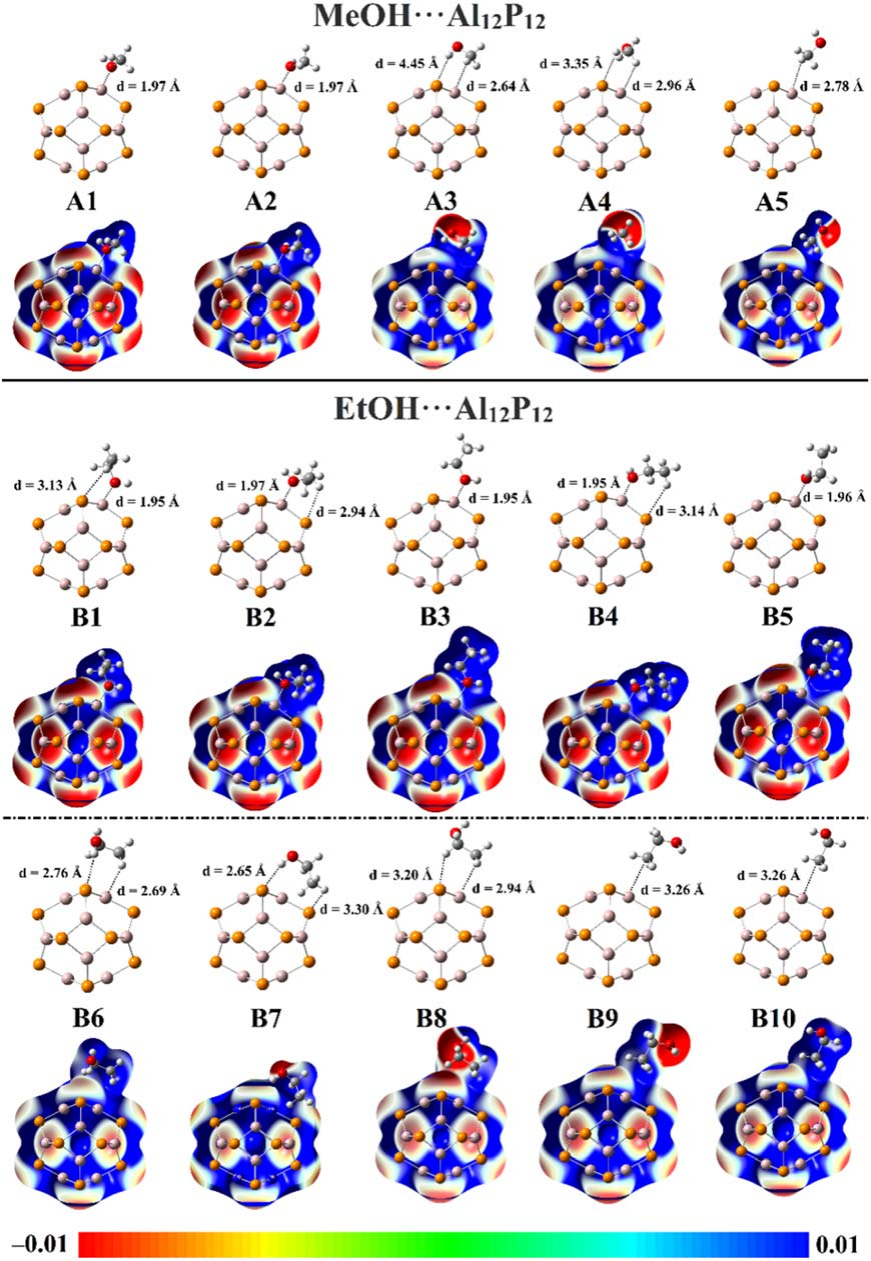
Optimized structures and MEP maps of the MeOH⋯ and EtOH⋯Al_12_P_12_ complexes within all plausible configurations. The intermolecular distances are in Å.

To gain an extensive comprehension of the adsorption process, the VOCs were placed on the surface of the Al_12_P_12_ nanocage. Geometrical optimization calculations were executed for the VOC⋯Al_12_P_12_ complexes within all plausible configurations (Fig. 2). No imaginary frequency was identified, confirming that the optimized structures are true minima. On the optimized VOC⋯Al_12_P_12_ complexes, the MEP maps were extracted and are displayed in Fig. 2. The adsorption (*E*_ads_) and interaction (*E*_int_) energies were accordingly assessed. Table 1 shows the computed *E*_ads_ and *E*_int_ values along with the intermolecular distances between the VOC molecule and Al_12_P_12_ nanocage.

**Table 1:** Calculated values of the adsorption energies (*E*_ads_, kcal mol^−1^) and the interaction energies (*E*_int_, kcal mol^−1^) of the optimized MeOH⋯ and EtOH⋯Al_12_P_12_ complexes within all plausible configurations in conjunction with intermolecular distances (*d*, Å)

Complexes	Configuration	Bond	*d*	*E*_ads_	*E*_int_
MeOH⋯Al_12_P_12_	A1	O⋯Al	1.97	−26.01	−30.76
	A2	O⋯Al	1.97	−25.37	−30.21
	A3	H⋯Al	2.64	−3.66	−3.93
		H⋯P	4.45		
	A4	H⋯Al	2.96	−3.66	−3.91
		H⋯P	3.35		
	A5	Al⋯H	2.78	−1.90	−1.97
EtOH⋯Al_12_P_12_	B1	O⋯Al	1.95	−27.23	−32.84
		H⋯P	3.13		
	B2	O⋯Al	1.97	−26.59	−32.45
		H⋯P	2.94		
	B3	O⋯Al	1.95	−27.03	−32.39
	B4	O⋯Al	1.95	−26.44	−32.17
		H⋯P	3.14		
	B5	O⋯Al	1.96	−26.21	−31.50
	B6	H⋯Al	2.69	−4.47	−4.91
		H⋯P	2.76		
	B7	H1⋯P1	2.65	−4.68	−4.84
		H2⋯P2	3.30		
	B8	H⋯Al	2.94	−2.81	−2.91
		H⋯P	3.20		
	B9	C⋯Al	3.26	−2.44	−2.52
	B10	C⋯Al	3.26	−2.43	−2.51

According to data presented in Table 1, the intermolecular distances were observed with values ranging from 4.45 to 1.97 and 3.30 to 1.95 Å for the optimized MeOH⋯ and EtOH⋯Al_12_P_12_ complexes, respectively. Notably, the VOC⋯Al_12_P_12_ complexes exhibited significant negative *E*_ads_ and *E*_int_ values, ensuring the efficacy of the Al_12_P_12_ nanocage toward adsorbing VOC molecules. The EtOH⋯Al_12_P_12_ complexes showed higher negative *E*_ads_ and *E*_int_ values relative to the MeOH⋯Al_12_P_12_ complexes. Numerically, *E*_ads_/*E*_int_ of the interactions within the MeOH⋯ and EtOH⋯Al_12_P_12_ complexes showed values ranging from −1.90/−1.97 to −26.01/−30.76 and from −2.43/−2.51 to −27.23/−32.84 kcal mol^−1^, respectively. It is worth noting that the selectivity of Al_12_P_12_ nanocage toward adsorbing EtOH over MeOH molecules is not guaranteed where the energy differences between MeOH⋯ and EtOH⋯Al_12_P_12_ complexes is about 2 kcal mol^−1^.

For the sake of comparison, more favorability was denoted in the case of configurations A1 ↔ A2 and B1 ↔ B5 within the MeOH⋯ and EtOH⋯Al_12_P_12_ complexes, respectively. In the abovementioned configurations, the investigated VOCs were adsorbed on the surface of the Al_12_P_12_ nanocage *via* the interactions of their O atoms and the nanocage's Al atom. This finding was in line with the ESP results (Fig. 2) that confirmed the predominant nucleophilic character around the O atom in the VOCs. It was noticeable that the most preferred MeOH⋯ and EtOH⋯Al_12_P_12_ complexes were denoted in the case of the configurations A1 and B1, which had *E*_ads_ values of −26.01 and −27.23 kcal mol^−1^, respectively.

### 2.3. SAPT calculations

**Fig. 3:**
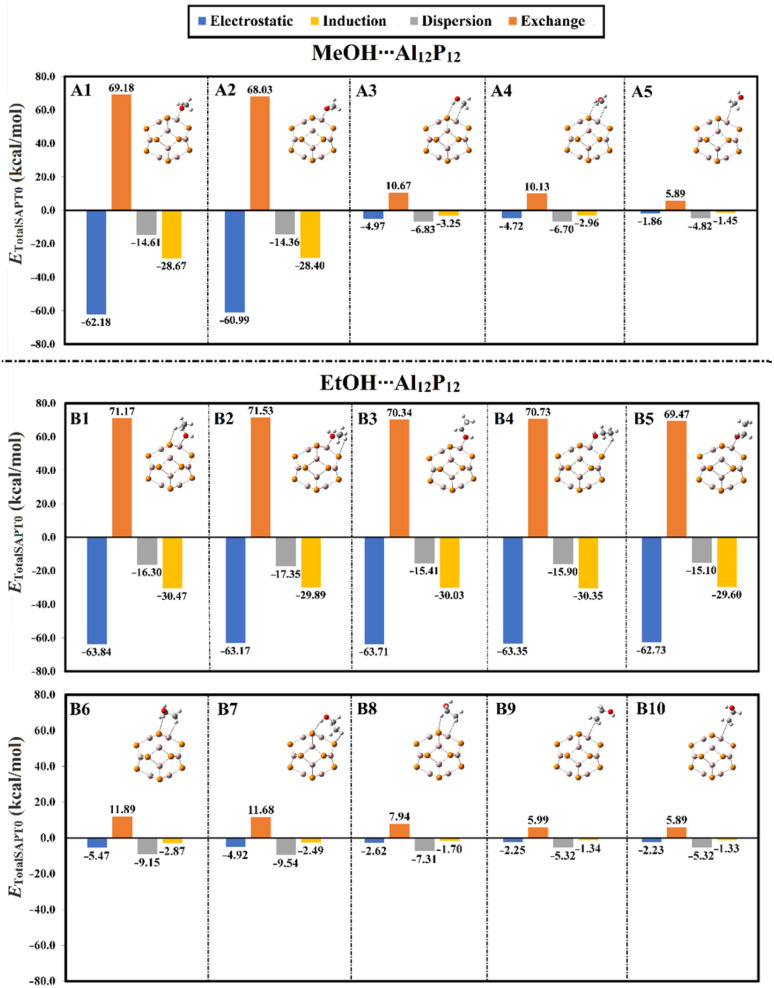
Graphical representation demonstrating the energetic components of total SAPT0 energies of the optimized MeOH⋯ and EtOH⋯Al_12_P_12_ complexes within all plausible configurations.

To unveil the contributions of the physical energetic components to the inspected adsorption process, SAPT analysis was carried out for the optimized MeOH⋯ and EtOH⋯Al_12_P_12_ complexes within all plausible configurations. Fig. 3 illustrates the four main components of the total SAPT0 energy, namely induction (*E*_ind_), electrostatic (*E*_elst_), exchange (*E*_exch_), and dispersion (*E*_disp_).

Looking at Fig. 3, the *E*_elst_, *E*_ind_, and *E*_disp_ exhibited negative values, revealing their favorable role as attractive forces between the interacted species within the inspected complexes. It is worth mentioning that the *E*_elst_ forces were dominant within the most preferable configurations of the VOC⋯Al_12_P_12_ complexes. Such findings could be attributed to the interaction of the electron-rich oxygen atom in MeOH and EtOH with the electron-deficient aluminium atom in the Al_12_P_12_ nanocage. For instance, the *E*_elst_ values of the configurations A1 and B1 were −62.18 and −63.84 kcal mol^−1^, respectively. For the other configurations (*i.e.*, A3 ↔ A5 and B6 ↔ B10), the *E*_disp_ component exhibited notable contributions to the overall attractive forces beyond the occurrence of the adsorptions process. While the *E*_exch_ component was found with positive values, ensuring its unfavorable contribution to the adsorption process of the VOCs onto the surface of the Al_12_P_12_ nanocage.

### 2.4. QTAIM and NCI analyses

**Fig. 4:**
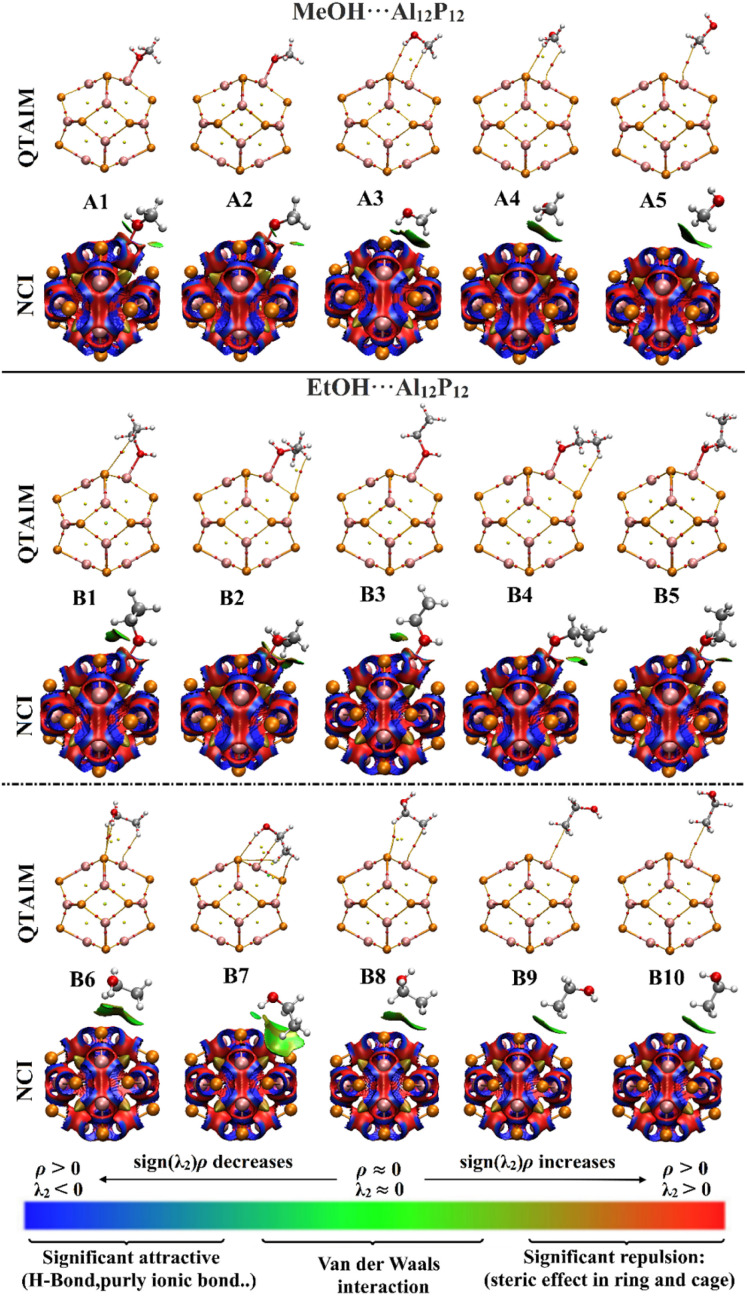
QTAIM and 3D NCI plots of the optimized MeOH⋯ and EtOH⋯Al_12_P_12_ complexes within all plausible configurations.

QTAIM and NCI index analyses were employed to unveil an in-depth elucidation of the nature and origin of the interactions within the investigated complexes (Fig. 4). As demonstrated in Fig. 4, the occurrence of the adsorption process within the optimized MeOH⋯ and EtOH⋯Al_12_P_12_ complexes was assured by the presence of bond paths (BPs) and bond critical points (BCPs) between the interacted species.

To better comprehend the interaction of VOCs with the Al_12_P_12_ nanocage, total energy density (*H*_b_), electron density (*ρ*_b_), Laplacian (∇^2^*ρ*_b_), kinetic electron density (*G*_b_), local potential electron energy density (*V*_b_), and the negative ratio of kinetic and potential electron energy density (−*G*_b_/*V*_b_) were computed at bond critical points and tabulated in Table 2. From the summarized data in Table 2, the positive ∇^2^*ρ*_b_ and *H*_b_ values asserted the closed-shell nature of the interactions within the complexes under investigation. For instance, the *H*_b_/∇^2^*ρ*_b_ values of the optimized MeOH⋯ and EtOH⋯Al_12_P_12_ complex within configuration A1 and B1 were 0.0040/0.3198 and 0.0051/0.3370 au, respectively.

**Table 2:** Topological features at BCPs of the optimized MeOH⋯ and EtOH⋯Al_12_P_12_ complexes within all the plausible configurations. All parameters are provided in au

Complexes	Configuration	Bond	*ρ*_b_	*H*_b_	∇^2^*ρ*_b_	*G*_b_	*V*_b_	−*G*_b_/*V*_b_
MeOH⋯Al_12_P_12_	A1	O⋯Al	0.0505	0.0040	0.3198	0.0759	−0.0719	1.0559
	A2	O⋯Al	0.0499	0.0042	0.3180	0.0753	−0.0711	1.0590
	A3	H⋯Al	0.0086	0.0001	0.0158	0.0040	−0.0040	1.0005
		H⋯P	0.0119	0.0008	0.0348	0.0079	−0.0071	1.1113
	A4	H⋯Al	0.0610	0.0001	0.1233	0.0037	−0.0036	1.0310
		H⋯P	0.0610	0.0008	0.1234	0.0078	−0.0069	1.1184
	A5	Al⋯H	0.0074	0.0002	0.0148	0.0035	−0.0032	1.0743
EtOH⋯Al_12_P_12_	B1	O⋯Al	0.0513	0.0051	0.3370	0.0792	−0.0741	1.0682
		H⋯P	0.0058	0.0010	0.0172	0.0033	−0.0024	1.4054
	B2	O⋯Al	0.0503	0.0039	0.3171	0.0754	−0.0716	1.0539
		H⋯P	0.0089	0.0013	0.0280	0.0057	−0.0043	1.3059
	B3	O⋯Al	0.0514	0.0047	0.3346	0.0789	−0.0742	1.0633
	B4	O⋯Al	0.0509	0.0051	0.3345	0.0785	−0.0734	1.0696
		H⋯P	0.0057	0.0009	0.0170	0.0033	−0.0024	1.4001
	B5	O⋯Al	0.0510	0.0045	0.3295	0.0779	−0.0733	1.0618
	B6	H⋯Al	0.0082	0.0002	0.0162	0.0038	−0.0036	1.0635
		H⋯P	0.0095	0.0010	0.0298	0.0064	−0.0054	1.1933
	B7	H1⋯P1	0.0115	0.0008	0.0340	0.0077	−0.0069	1.1193
		H2⋯P2	0.0050	0.0007	0.0144	0.0029	−0.0021	0.7427
	B8	H⋯Al	0.0067	0.0001	0.0137	0.0033	−0.0031	1.0446
		H⋯P	0.0052	0.0009	0.0153	0.0029	−0.0020	1.4400
	B9	C⋯Al	0.0064	0.0001	0.0137	0.0033	−0.0032	0.9681
	B10	C⋯Al	0.0065	0.0001	0.0138	0.0033	−0.0032	0.9684

Looking at 3D NCI isosurfaces displayed in Fig. 4, various types of interactions are highlighted by different colored isosurfaces; blue demonstrates a stronger hydrogen bond, green represents van der Waals interactions, and red confirms steric effects. The existence of blue-green colored isosurfaces between VOCs and the surface of the Al_12_P_12_ nanocage within the investigated complexes shed light on the propensity of the Al_12_P_12_ nanocage toward sensing the inspected VOCs.

### 2.5. Electronic parameters

**Fig. 5:**
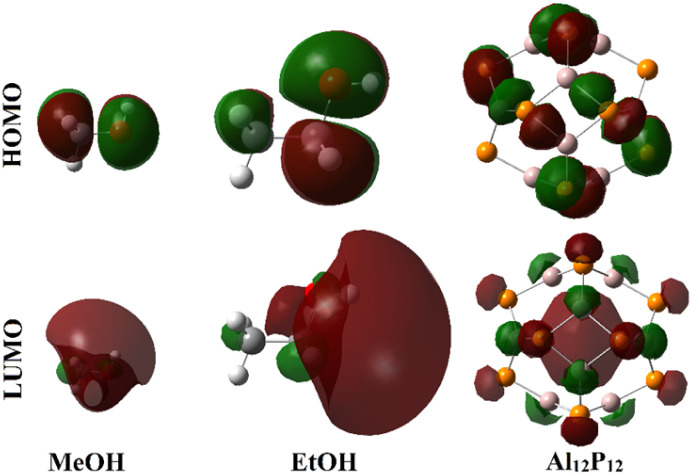
Plots of the distribution patterns of HOMO and LUMO of the MeOH, EtOH, and Al_12_P_12_ monomers.

**Fig. 6:**
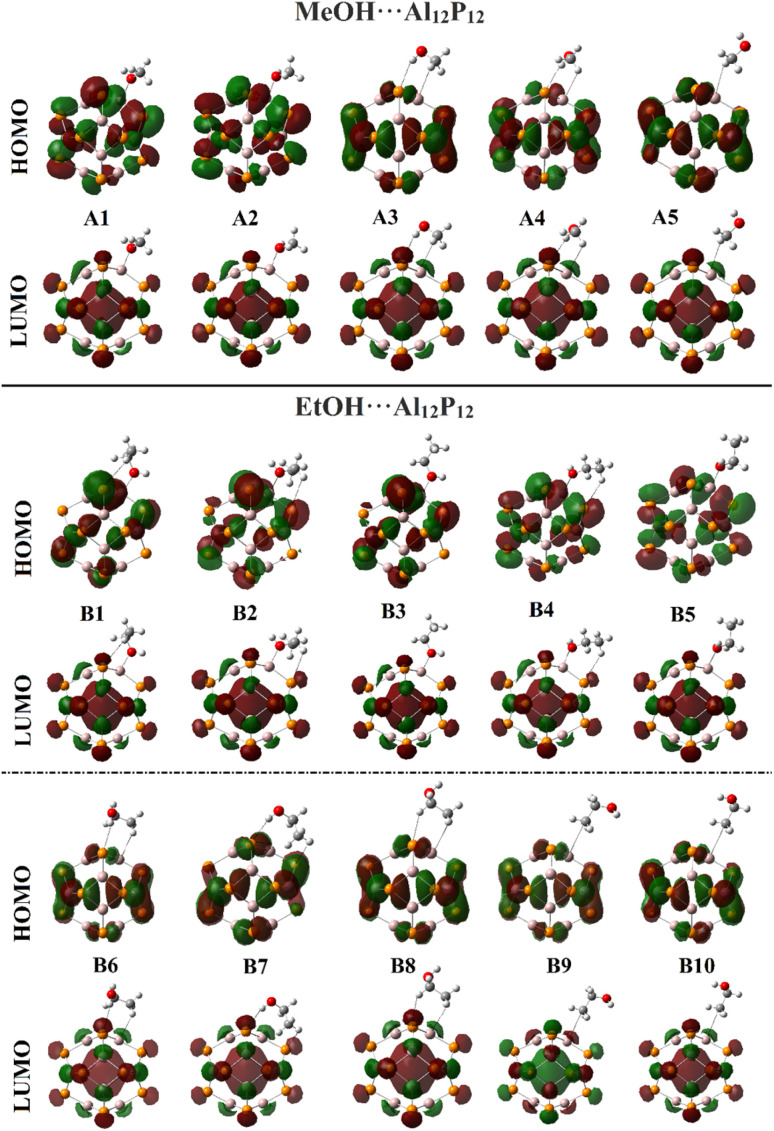
Plots of the distributions patterns of HOMO and LUMO of the optimized MeOH⋯ and EtOH⋯Al_12_P_12_ complexes in all the plausible configurations.

With the incorporation of frontier molecular orbital (FMO) theory, the electronic parameters and the distribution of the molecular orbitals were outlined for the VOCs and Al_12_P_12_ before and after the adsorption process. In this regard, the energies of the highest occupied molecular orbitals (*E*_HOMO_), the lowest unoccupied molecular orbitals (*E*_LUMO_), Fermi level (*E*_FL_), and energy gap (*E*_gap_) were determined to unveil the capability of the inspected systems within monomeric and complex forms to donate and accept electrons (Table 3). Fig. 5 and 6 illustrate the distribution patterns of the molecular orbitals of the studied systems within the monomeric and complex forms, respectively.

**Table 3:** Computed electronic parameters (in eV) of the optimized VOCs and Al_12_P_12_ nanocage within the monomeric and complex forms

System	Configuration	*E*_HOMO_	*E*_FL_	*E*_LUMO_	*E*_gap_
MeOH		−9.577	−4.523	0.531	10.108
EtOH		−9.447	−4.472	0.502	9.949
Al_12_P_12_		−7.755	−5.389	−3.024	4.731
MeOH⋯Al_12_P_12_	A1	−7.430	−5.063	−2.695	4.735
	A2	−7.424	−5.054	−2.683	4.742
	A3	−7.842	−5.473	−3.104	4.737
	A4	−7.843	−5.476	−3.109	4.735
	A5	−7.766	−5.402	−3.038	4.727
EtOH⋯Al_12_P_12_	B1	−7.398	−5.029	−2.660	4.738
	B2	−7.400	−5.036	−2.672	4.728
	B3	−7.393	−5.024	−2.654	4.739
	B4	−7.384	−5.020	−2.656	4.729
	B5	−7.389	−5.020	−2.651	4.738
	B6	−7.836	−5.469	−3.101	4.736
	B7	−7.851	−5.484	−3.118	4.733
	B8	−7.723	−5.359	−2.994	4.729
	B9	−7.757	−5.395	−3.034	4.724
	B10	−7.757	−5.393	−3.030	4.726

As illustrated in Fig. 5, the HOMO and LUMO distribution patterns were observed over the electronegative and electropositive regions of the studied VOCs. Considering the Al_12_P_12_ nanocage, the P and Al atoms were generally noticed with distributions of HOMO and LUMO orbitals, respectively. Following the interactions of the adopted VOCs with Al_12_P_12_, redistribution of HOMO and LUMO orbitals was denoted, highlighting the occurrence of the adsorption process (Fig. 6). On the investigated complexes, the HOMO and LUMO levels were found over the Al_12_P_12_ nanocage.

Upon the listed data in Table 3, notable changes in the *E*_HOMO_, *E*_FL_, *E*_LUMO_, and *E*_gap_ values following the adsorption of the VOCs on the Al_12_P_12_ nanocage were denoted, confirming the occurrence of the adsorption process. For example, the *E*_HOMO_ of the pure Al_12_P_12_ nanocage showed a value of −7.755 eV and changed to −7.430 and −7.398 eV after adsorbing MeOH and EtOH within the configurations A1 and B1, respectively. An apparent alteration in the *E*_gap_ values of the studied systems was also detected, outlining the prominent effect of the adsorption process of the MeOH and EtOH on the surface of the Al_12_P_12_ nanocage. As an illustration, the *E*_gap_ value of pure Al_12_P_12_ nanocage was 4.731 eV, which changed to 4.735 and 4.738 eV following the adsorption process within the configurations A1 and B1 of the MeOH⋯ and EtOH⋯Al_12_P_12_ complexes, respectively.

### 2.6. Global reactivity descriptors

In an attempt to clarify the effect of the adsorption process of the MeOH and EtOH molecules on the Al_12_P_12_ nanocage, global reactivity descriptors of the monomeric and complex forms of the studied systems were evaluated. Numerous parameters, comprising ionization potential (IP), electron affinity (EA), chemical potential (*μ*), global hardness (*η*), global softness (*S*), electrophilicity index (*ω*), and work function (*Φ*), were calculated and are compiled in Table 4.

**Table 4:** Global indices descriptors of the monomeric and complex forms of the investigated VOCs and Al_12_P_12_ nanocage

System	Configuration	IP (eV)	EA (eV)	*μ* (eV)	*η* (eV)	*S* (eV^−1^)	*ω* (eV)	*Φ* (eV)
MeOH		9.577	−0.531	−4.523	5.054	0.198	2.024	4.523
EtOH		9.447	−0.502	−4.472	4.975	0.201	2.010	4.472
Al_12_P_12_		7.755	3.024	−5.389	2.366	0.423	6.139	5.389
MeOH⋯Al_12_P_12_	A1	7.430	2.695	−5.063	2.368	0.422	5.413	5.063
	A2	7.424	2.683	−5.054	2.371	0.422	5.386	5.054
	A3	7.842	3.104	−5.473	2.369	0.422	6.323	5.473
	A4	7.843	3.109	−5.476	2.367	0.422	6.334	5.476
	A5	7.766	3.038	−5.402	2.364	0.423	6.173	5.402
EtOH⋯Al_12_P_12_	B1	7.398	2.660	−5.029	2.369	0.422	5.338	5.029
	B2	7.400	2.672	−5.036	2.364	0.423	5.364	5.036
	B3	7.393	2.654	−5.024	2.370	0.422	5.326	5.024
	B4	7.384	2.656	−5.020	2.364	0.423	5.329	5.020
	B5	7.389	2.651	−5.020	2.369	0.422	5.319	5.020
	B6	7.836	3.101	−5.469	2.368	0.422	6.315	5.469
	B7	7.851	3.118	−5.484	2.367	0.423	6.355	5.484
	B8	7.723	2.994	−5.359	2.364	0.423	6.072	5.359
	B9	7.757	3.034	−5.395	2.362	0.423	6.163	5.395
	B10	7.757	3.030	−5.393	2.363	0.423	6.155	5.393

Relying on the summarized data in Table 4, substantial alterations in the values of global reactivity descriptors of the Al_12_P_12_ before and following the adsorption process were observed, outlining the influence of the adsorption process on the reactivity character of the utilized nanocage. For instance, the IP value of pure Al_12_P_12_ nanocage was 7.755 eV and altered to 7.430 and 7.398 eV following interaction with VOCs within configurations A1 and B1, respectively. Apparently, upward and downward shifts in the *η* and *S* values were observed following the adsorption process. As numerical evidence, *η* of pure Al_12_P_12_ nanocage was 2.366 eV and boosted to 2.368 and 2.369 eV for the configurations A1 and B1, respectively. Remarkably, the alterations in work function affirmed the potency of Al_12_P_12_ nanocage as a promising sensing material for MeOH and EtOH molecules.

### 2.7. DOS analysis

**Fig. 7:**
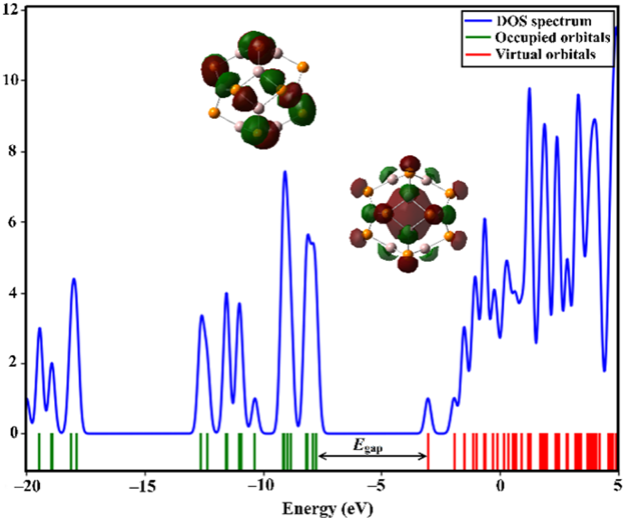
The DOS plot for the pure Al_12_P_12_ nanocage before the adsorption process.

**Fig. 8:**
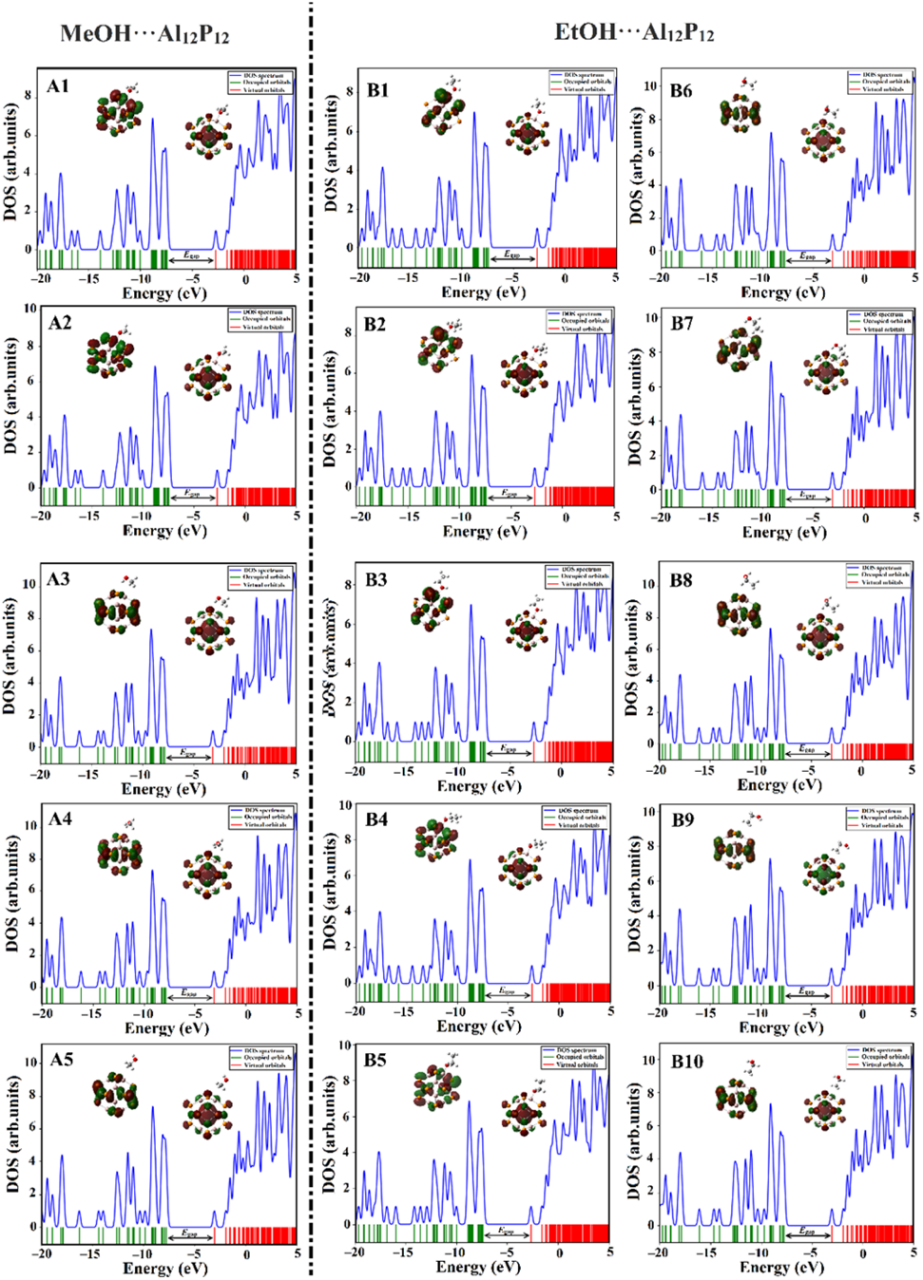
The DOS plots for Al_12_P_12_ nanocage following the adsorption process of the optimized MeOH⋯ and EtOH⋯Al_12_P_12_ complexes within all plausible configurations.

DOS analysis was executed to unveil the change in the electronic characteristics of the A_12_P_12_ nanocage after the adsorption of MeOH and EtOH molecules. Fig. 7 and 8 depict the DOS plots of the Al_12_P_12_ nanocage before and following the adsorption process within all plausible configurations of the VOC⋯Al_12_P_12_ complexes, respectively.

Notably, new peaks were detected by comparing the DOS plots of the Al_12_P_12_ nanocage before and following the adsorption process (Fig. 7 and 8, respectively). This result outlined the influential effect of adsorbing VOCs on the electrical characteristics of the Al_12_P_12_ nanocage. For instance, additional peaks in the valence region were denoted from −13.50 to −18.00 eV in the DOS plots of almost all studied configurations. The variations in energy gap values were scrutinized for all studied complexes, confirming the ability of the studied Al_12_P_12_ nanocage to sense VOCs with disparate efficiencies.

### 2.8. Thermodynamic parameters

To gain a thorough understanding of the adsorption process within the MeOH⋯ and EtOH⋯Al_12_P_12_ complexes, thermodynamic parameters (*i.e.*, changes in enthalpy (Δ*H*), Gibbs free energy (Δ*G*), and entropy (Δ*S*)) were calculated for all the investigated complexes, and the outcomes are presented in Table 5.

**Table 5:** Thermodynamic parameters of the optimized MeOH⋯ and EtOH⋯Al_12_P_12_ complexes within all plausible configurations are in kcal mol^−1^

Complex	Configuration	Δ*G*	Δ*H*	Δ*S*
MeOH⋯Al_12_P_12_	A1	−13.00	−24.32	−0.038
	A2	−12.75	−23.74	−0.037
	A3	6.03	−2.64	−0.029
	A4	6.00	−2.65	−0.029
	A5	5.98	−0.86	−0.023
EtOH⋯Al_12_P_12_	B1	−14.33	−25.71	−0.038
	B2	−13.43	−24.99	−0.039
	B3	−14.37	−25.47	−0.037
	B4	−13.99	−24.97	−0.037
	B5	−13.76	−24.64	−0.036
	B6	5.99	−3.47	−0.032
	B7	5.96	−3.69	−0.032
	B8	6.61	−1.79	−0.028
	B9	5.68	−1.32	−0.023
	B10	6.42	−1.27	−0.026

According to the data listed in Table 5, the negative values of Δ*G* confirm the spontaneity of the adsorption process within the most preferable configurations of the VOC⋯Al_12_P_12_ complexes. Significantly, the exothermic nature was noticed and confirmed by negative Δ*H* values for the optimized VOC⋯Al_12_P_12_ complexes within all inspected configurations. Remarkably, small negative Δ*S* values were obtained, unveiling the randomness in all studied complexes. In alignment with the *E*_ads_ results, configurations A1 and B1 of the VOC⋯Al_12_P_12_ complexes showed the highest negative values of thermodynamic energetic quantities. For instance, configuration B1 was thermodynamically stable with negative Δ*G*, Δ*H*, and Δ*S* values of −14.33, −25.71, and −0.038 kcal mol^−1^, respectively. The abovementioned observations outlined the proficiency of Al_12_P_12_ nanocage toward sensing the studied VOCs.

### 2.9. IR and Raman spectra

**Fig. 9:**
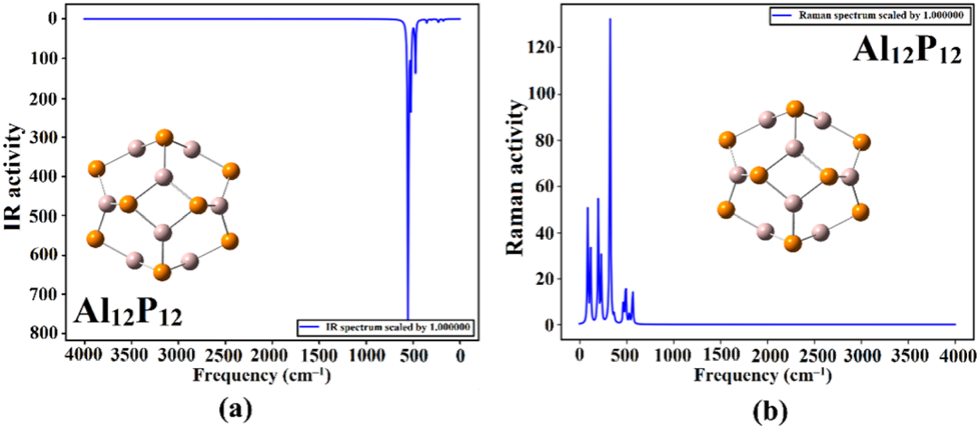
Plots of (a) IR and (b) Raman spectra of pure Al_12_P_12_ nanocage.

**Fig. 10:**
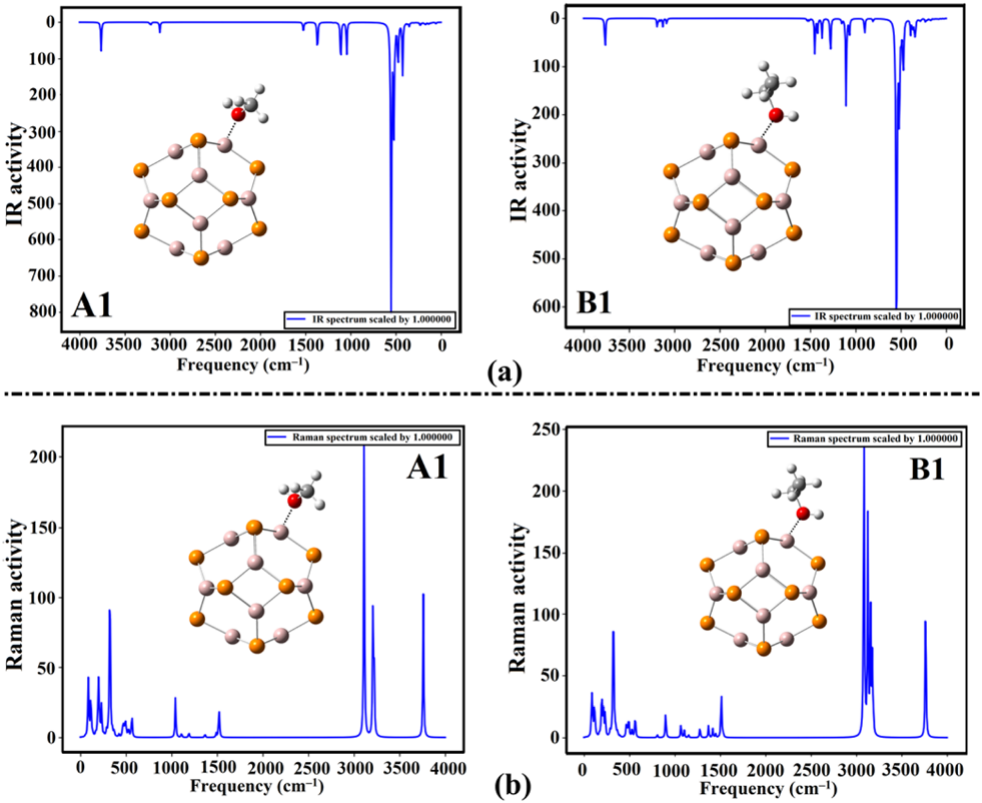
Plots of (a) IR and (b) Raman spectra of the optimized MeOH⋯ and EtOH⋯Al_12_P_12_ complexes within configurations A1 and B1.

To ensure the occurrence of the adsorption process of the MeOH and EtOH molecules on the Al_12_P_12_ nanocage, IR and Raman spectra were extracted for pure Al_12_P_12_ nanocage (Fig. 9) and the optimized MeOH⋯ and EtOH⋯Al_12_P_12_ complexes within all plausible configurations (Fig. S1 and S2†). Fig. 10 represents plots of IR and Raman spectra of the optimized MeOH⋯ and EtOH⋯Al_12_P_12_ complexes within configurations A1 and B1 as an illustration.

As depicted in Fig. 9(a), the most noticeable IR band in pure Al_12_P_12_ nanocage was ascribed to Al⋯P stretching that appeared at 550 cm^−1^. Following the adsorption of VOCs on the Al_12_P_12_ nanocage, the Al⋯P stretching vibrations were denoted with distinct changes in the intensities within the studied complexes (Fig. 10(a)). Obviously, new additional bands appeared in all studied complexes, affirming the substantial adsorption of MeOH and EtOH on Al_12_P_12_ nanocage (Fig. S1†).

Similarly, significant alterations in the Raman spectra were noticed between the pure and complex forms of the Al_12_P_12_ nanocage (Fig. 10(b)). Overall, the notable difference in the IR and Raman spectra (Fig. S1 and S2,† respectively) announced the potential efficacy of the Al_12_P_12_ nanocage in detecting MeOH and EtOH molecules.

### 2.10. Recovery time

Recovery time (*τ*) values were computed for the optimized MeOH⋯ and EtOH⋯Al_12_P_12_ complexes within all plausible configurations toward a better comprehension of the required time for the VOC to separate from the surface of the Al_12_P_12_ nanocage (Table 6).

**Table 6:** The calculated *τ* values of the optimized MeOH⋯ and EtOH⋯Al_12_P_12_ complexes within all plausible configurations

Complex	Configuration	*τ* (ms)
MeOH⋯Al_12_P_12_	A1	1.09 × 10^10^
	A2	3.72 × 10^9^
	A3	4.78 × 10^−7^
	A4	4.78 × 10^−7^
	A5	2.46 × 10^−8^
EtOH⋯Al_12_P_12_	B1	8.54 × 10^10^
	B2	2.91 × 10^10^
	B3	6.10 × 10^10^
	B4	2.26 × 10^10^
	B5	1.53 × 10^10^
	B6	1.87 × 10^−6^
	B7	2.66 × 10^−6^
	B8	1.14 × 10^−7^
	B9	6.11 × 10^−8^
	B10	6.01 × 10^−8^

Relying on the recorded data in Table 6, the *τ* findings were directly proportional to the *E*_ads_ values, revealing that the time required for VOC to dissociate from the adsorbent surface increased with augmenting *E*_ads_ value. For the sake of clarification, the highest negative *E*_ads_ values were ascribed to the EtOH⋯Al_12_P_12_ complexes, which were denoted with longer *τ* values compared to MeOH⋯Al_12_P_12_ complexes. As numerical evidence, the configurations A1 and B1 of the MeOH⋯ and EtOH⋯Al_12_P_12_ complexes possessed the most pronounced negative *E*_ads_ values of −26.01 and −27.23 kcal mol^−1^ accompanied with *τ* values of 1.09 × 10^10^ and 8.54 × 10^10^ ms, respectively. Consequently, the Al_12_P_12_ nanocage was considered an appropriate sensor for MeOH and EtOH molecules.

## 3. Computational methods

The adsorption amplitude of VOCs (*i.e.*, MeOH and EtOH) over the Al_12_P_12_ nanocage was fully investigated using a plethora of DFT computations with the aid of the Gaussian 09 package.^43^ For the investigated systems, the geometrical optimization accompanied by frequency computations was carried out at the M06-2X^44^ method simultaneously with a 6-31+G* basis set.

To illustrate the nucleophilic and electrophilic characters of the MeOH, EtOH, and Al_12_P_12_, the electrostatic potential (ESP) analysis was conducted. Using an electron density envelope of 0.002 au,^45^ surface electrostatic potential extrema (*V*_s,min_/*V*_s,max_) and molecular electrostatic potential (MEP) maps were evaluated and extracted to provide numerical and graphical explanations for the investigated systems, respectively. The *V*_s,min_/*V*_s,max_ were obtained by adopting the Multiwfn 3.7 software.^46^

The efficacy of Al_12_P_12_ nanocage toward adsorbing VOCs was thoroughly determined in terms of adsorption (*E*_ads_) and interaction (*E*_int_) energies. For the VOC⋯Al_12_P_12_ complexes, *E*_ads_ and *E*_int_ were computed utilizing the counterpoise corrected (CC) method to eliminate the basis set superposition error (BSSE),^47^ relying on eqn (1) and (2), respectively.

1 *E*_ads_ = *E*_VOC⋯Al12P12_ − (*E*_VOC_ + *E*_Al_12_P_12__) + *E*_BSSE_

2 *E*_int_ = *E*_VOC⋯Al_12_P_12__ − (*E*_VOC in complex_ + *E*_Al_12_P_12_ in complex_) + *E*_BSSE_

where *E*_VOC⋯Al_12_P_12__, *E*_VOC_, and *E*_Al_12_P_12__ represent the energies of investigated complexes, isolated VOCs, and Al_12_P_12_ nanocage, respectively. Whereas the *E*_VOC in complex_ and *E*_Al_12_P_12_ in complex_ identify the energies of the MeOH/EtOH molecules and Al_12_P_12_ nanocage based on their coordinates in the complex form.

Moreover, SAPT analysis was performed employing the SAPT0 level of truncation using the PSI4 code.^48^ In the context of SAPT, the total energy (*E*^SAPT0^) was divided into *E*_ind_, *E*_elst_, *E*_exch_, and *E*_disp_. *E*^SAPT0^ was evaluated utilizing eqn (3).^49–51^

3 *E*^SAPT0^ = *E*_elst_ + *E*_exch_ + *E*_ind_ + *E*_disp_

Wavefunction analyses, including NCI index and QTAIM, were executed for the VOC⋯Al_12_P_12_ complexes using the Multiwfn 3.7 software^46^ and visualized by the Visual Molecular Dynamics program.^52^ With the inclusion of QTAIM, the BPs and BCPs between the interacted species were extracted. The topological parameters were evaluated for all the studied complexes. Considering the NCI index, the 3D colored isosurfaces were extracted depending on the sign(*λ*_2_)*ρ* varying between blue (−0.035 au) to red (0.020 au).

Toward obtaining an adequate illustration of the electronic properties before and following the adsorption process, the FMO theory was implemented. In this regard, the HOMO/LUMO distribution patterns were plotted for the monomeric and complex forms. Similarly, HOMO/LUMO energies (*E*_HOMO_/*E*_LUMO_) were determined. Upon the obtained *E*_HOMO_ and *E*_LUMO_ values, the *E*_gap_ and *E*_FL_ values were determined as follows:4
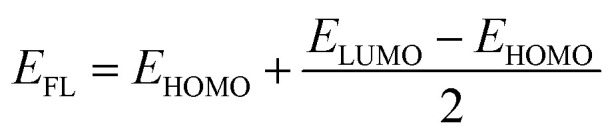

5 *E*_gap_ = *E*_LUMO_ − *E*_HOMO_

Based on the data obtained from FMO, the IP and EA were predestined based on eqn (6) and (7).

6 IP ≈ −*E*_HOMO_

7 EA ≈ −*E*_LUMO_

By applying Koopman's theorem,^53^ the chemical reactivity descriptors of molecules could be predicted based on quantum mechanical descriptors. Accordingly, *η*, *ω*, *S*, and *μ* were calculated utilizing eqn (8)–(11).8
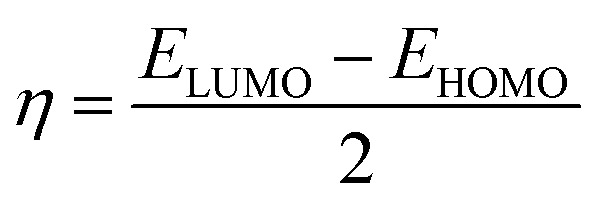
9
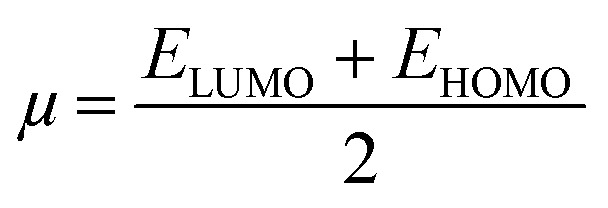
10
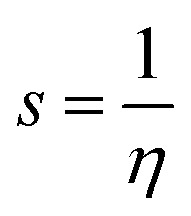
11
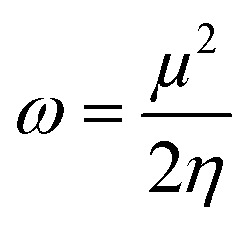


Afterwards, the *Φ* was calculated to determine the sensing ability of the studied nanocages using eqn (12),^54^ where *V*_el(+∞)_ identifies the electrostatic potential far from the nanocage surface that was postulated to be ≈0.

12 *Φ* = *V*_el(+∞)_ − *E*_FL_

To elucidate the influence of the adsorption process on the electronic properties of the utilized nanocage, density of states (DOS) plots were extracted within an energy range of −20 to +5 eV before and following the adsorption process based on eqn (13) employing the GaussSum software.^55^13
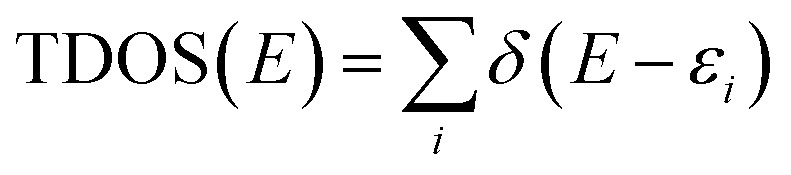
where *ε* and *δ* represent the eigenvalue set of single-particle Hamilton and Dirac delta function, respectively.

To assess the thermodynamic parameters of the inspected complexes, Δ*H*, Δ*G*, and Δ*S* were evaluated based on frequency calculations as follows:

14 Δ*M* = *M*_VOC⋯Al_12_P_12__ − (*M*_VOC_ + *M*_Al_12_P_12__) + *E*_BSSE_

15 Δ*S* = −(Δ*G* − Δ*H*)/*T*

whereas *M* indicates the quantity of *G* and *H*. The *M* of investigated complexes, VOCs, and nanocage were represented by *M*_VOC⋯Al_12_P_12__, *M*_VOC_, and *M*_Al_12_P_12__, respectively. *T* refers to temperature with a value of 298.15 K. Upon frequency computations, plots of IR and Raman spectra were extracted with the aid of GaussSum software.^55^ Recovery time (*τ*) was subsequently calculated to evaluate the feasibility of the desorption process within the complexes understudy using formula (16),^56,57^ where *v*_0_ and *K* represent the attempt frequency with a value of 10^12^ s^−1^ and Boltzmann's constant, respectively.

16 *τ* = *v*^−1^_0_ exp(−Δ*E*_ads_/*KT*)

## 4. Conclusions

The sensitivity of Al_12_P_12_ nanocage toward sensing MeOH and EtOH molecules was investigated in all plausible configurations utilizing numerous DFT calculations. The ESP outcomes unveiled the existence of evident nucleophilic and electrophilic regions over the surface of the MeOH and EtOH molecules, in particular around the O and C/H atoms, respectively. In comparison, the Al_12_P_12_ nanocage was observed with nucleophilic and electrophilic regions surrounding P and Al atoms, respectively. According to the energetic findings, the adsorption process showed higher preferability in the case of the EtOH⋯Al_12_P_12_ complexes compared to the MeOH⋯Al_12_P_12_ complexes with *E*_int_/*E*_ads_ values up to −32.84/−27.23 and −30.76/−26.01 kcal mol^−1^, respectively. SAPT affirmations revealed the *E*_elst_ forces with immense contributions to the attractive forces within the most preferable configurations of the VOC⋯Al_12_P_12_ complexes. QTAIM and NCI index results assured the noncovalent nature of the interaction within the studied complexes. The noticeable changes in molecular orbitals distribution patterns of MeOH/EtOH/Al_12_P_12_ nanocage, the electronic parameters, and the global reactivity descriptors highlighted the occurrence of the adsorption of VOCs on Al_12_P_12_ nanocage. Remarkably, thermodynamic parameters substantiated the exothermic character of the VOC⋯Al_12_P_12_ complexes within all plausible configurations. Thermodynamic parameters were denoted with negative values, demonstrating the spontaneous exothermic nature of the most investigated complexes. The appearance of new peaks in DOS plots confirmed the occurrence of the adsorption process between the studied VOCs and Al_12_P_12_ nanocage. Based on IR and Raman spectra findings, the occurrence of the adsorption process was ensured by the appearance of new bands in IR and Raman spectra. Recovery time results addressed the Al_12_P_12_ nanocage as an appropriate sensor for MeOH and EtOH molecules with *τ* values ranging from 6.11 × 10^−8^ to 8.54 × 10^10^ ms. The emerging findings would provide a comprehensive insight into the efficiency of the Al_12_P_12_ nanocage in detecting VOCs, especially for MeOH and EtOH molecules.

## Author contributions

Conceptualization, Mahmoud A. A. Ibrahim and Tamer Shoeib; methodology, Mahmoud A. A. Ibrahim, Nayra A. M. Moussa, and Ahmed M. Awad; software, Mahmoud A. A. Ibrahim; formal analysis, Manar H. A. Hamad; investigation, Manar H. A. Hamad and Nayra A. M. Moussa; resources, Mahmoud A. A. Ibrahim, Shaban R. M. Sayed, Omar H. Abd-Elkader and Tamer Shoeib; data curation, Manar H. A. Hamad; writing—original draft preparation, Manar H. A. Hamad; writing—review and editing, Mahmoud A. A. Ibrahim, Nayra A. M. Moussa, Shaban R. M. Sayed, Omar H. Abd-Elkader, Muhammad Naeem Ahmed, Ahmed M. Awad, and Tamer Shoeib; visualization, Manar H. A. Hamad and Muhammad Naeem Ahmed; supervision, Mahmoud A. A. Ibrahim; project administration, Mahmoud A. A. Ibrahim, Nayra A. M. Moussa and Tamer Shoeib. All authors have read and agreed to the published version of the manuscript.

## Conflicts of interest

There are no conflicts to declare.

## Supplementary Material

RA-014-D4RA01828A-s001
