# Supplementary material for: Aluminium phosphide (Al12P12) nanocage as a potential sensor for volatile organic compounds: A DFT study
Source: RSC Adv. 2024 Apr 29;14(20):13915–25. doi: 10.1039/d4ra01828a (PMC11056777; doi:10.1039/d4ra01828a)
Supplement: RA-014-D4RA01828A-s001 [file RA-014-D4RA01828A-s001.pdf]

Electronic Supplementary Material (ESI) for RSC Advances.
This journal is © The Royal Society of Chemistry 2024

Supporting Information

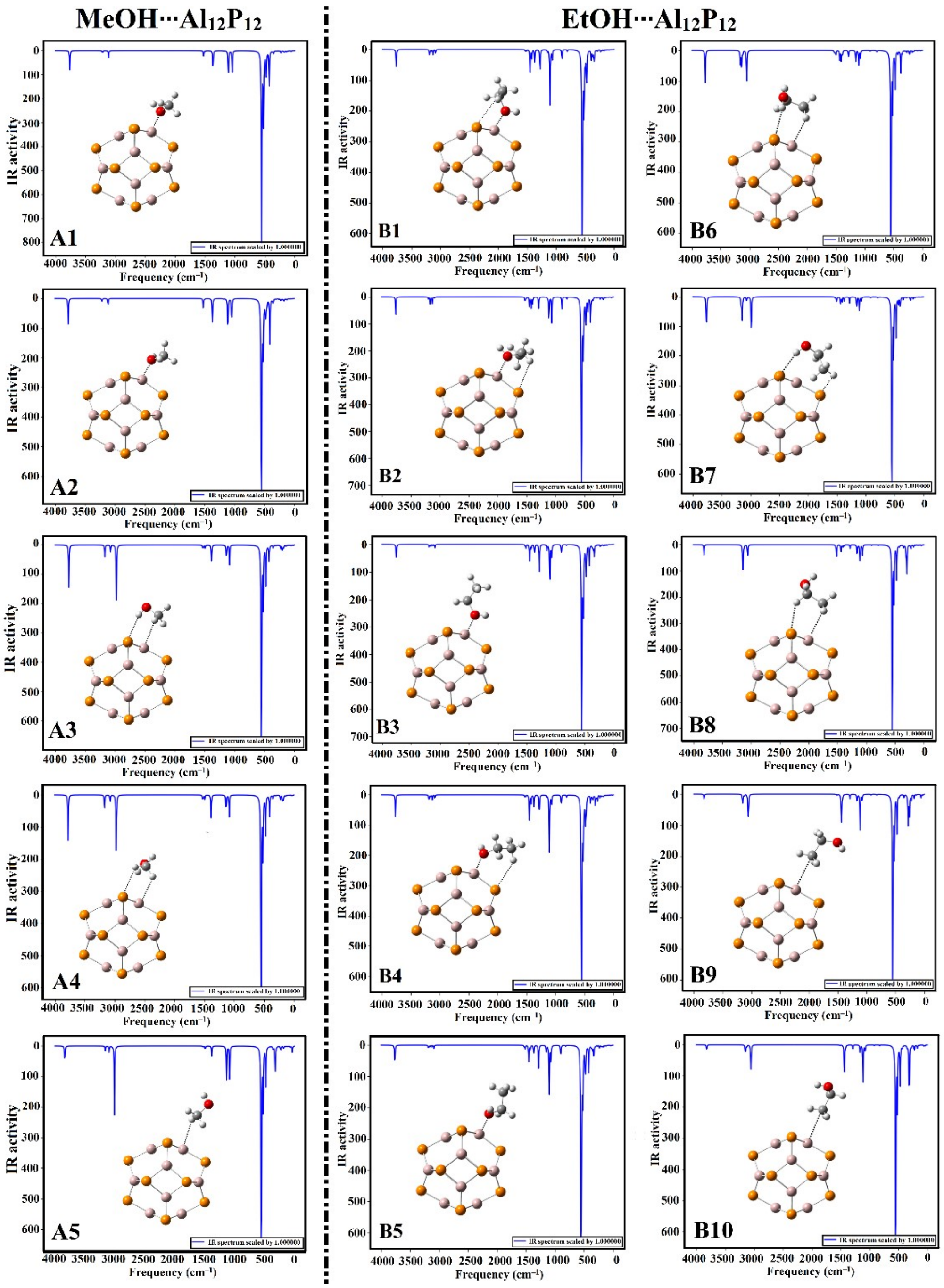

Figure S1. IR spectra of the optimized MeOH··· and EtOH···$Al_{12}P_{12}$ complexes within all plausible configurations.

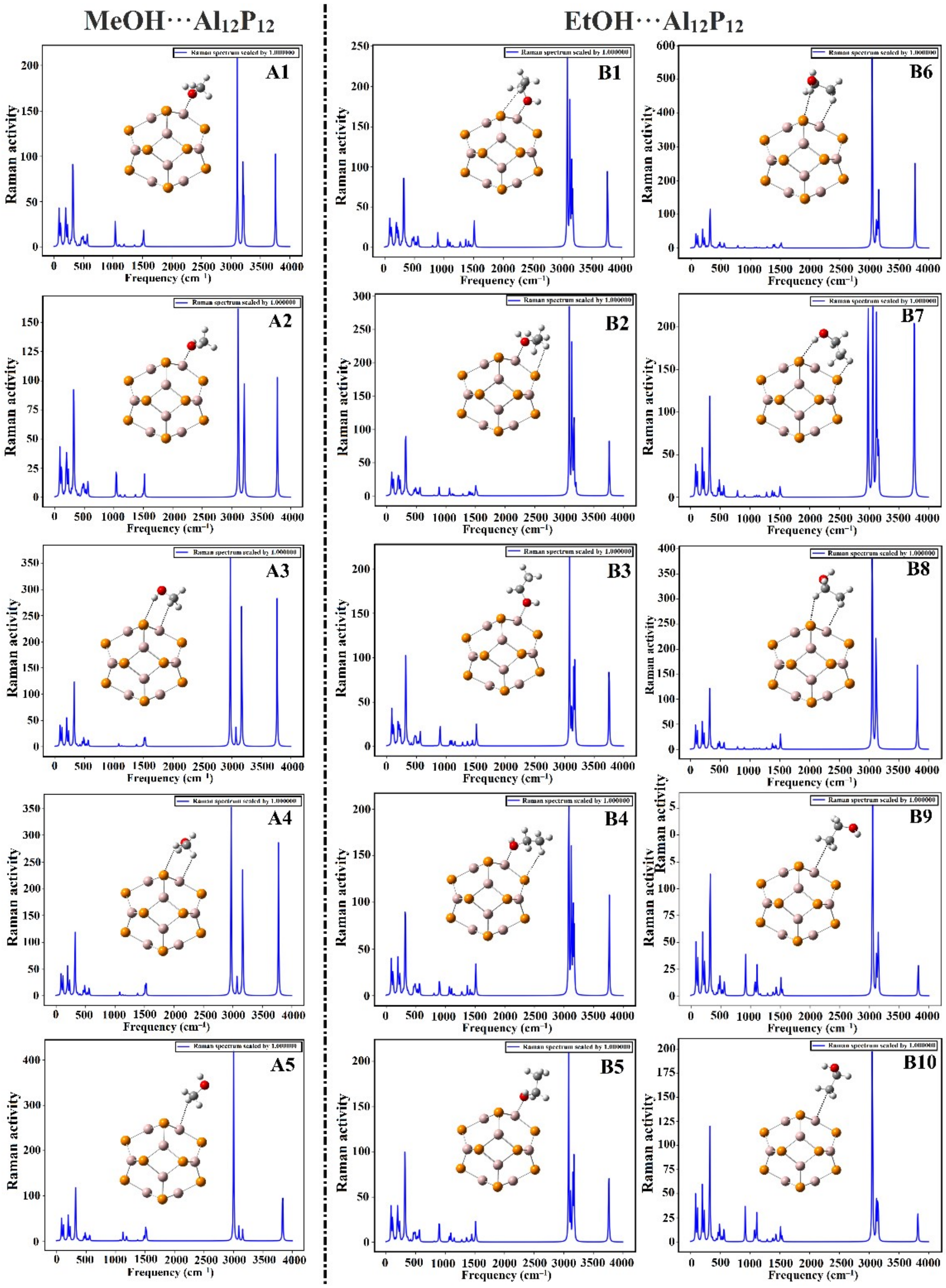

Figure S2. Raman spectra of the optimized MeOH··· and EtOH···$Al_{12}P_{12}$ complexes within all plausible configurations.

Cartesian atomic coordinates and Absolute Energies

Complex A1

Absolute Energy = -7121.233919 au.

Cartesian atomic coordinates:

P	-2.39156000	-1.82906900	-1.91106300
P	1.68516800	-1.81848200	-2.80550200
P	0.32969300	-3.49204500	0.78020200
P	-3.06327100	-1.17207000	1.50210200
P	-1.33907300	2.38457300	2.85244500
P	-3.01664700	2.18759900	-0.96927800
P	-0.05347300	1.11923400	-3.71972600
P	3.40728700	1.73968000	-1.45780700
P	3.32551800	-1.61349700	1.00608200
P	0.38984000	-0.56379200	3.76171800
P	2.76061000	2.37215800	2.00174000
P	0.01013000	4.00276000	-0.73115300
Al	2.07246800	3.16810500	-0.11178900
Al	0.82876800	1.53618400	2.85220400
Al	-1.48186400	2.89394500	0.63687900
Al	-1.55623000	0.07805200	2.66372500
Al	-0.48072900	-0.97655100	-2.80925400
Al	1.89314200	0.49506200	-2.60192100
Al	3.22621700	0.62145400	0.55771500
Al	-2.93160400	-0.03843800	-0.52589500
Al	-1.55880100	-2.36846000	0.19273200
Al	1.28633900	-1.82655400	2.09615300
Al	1.81478300	-2.34476800	-0.59736800
Al	-0.95631000	2.36535300	-2.04244200
O	3.16203800	4.80461600	-0.12214600
C	3.46674300	5.47936500	-1.36441100
H	4.13815300	4.86277000	-1.96563900
H	3.90515200	6.45116600	-1.13705900
H	2.51061700	5.60996000	-1.87160700
H	3.94503700	4.71574000	0.44909100

Complex A2

**Absolute Energy = -7121.232922 au.**			
**Cartesian atomic coordinates:**			
P	-2.57090600	-2.14322300	-1.71892000
P	1.43806100	-1.95072500	-2.86002300
P	0.38928600	-3.61640500	0.82850500
P	-3.07108000	-1.45708000	1.71692300
P	-1.45398400	2.20399800	2.90689000
P	-3.33946100	1.85565600	-0.80562800
P	-0.49420600	0.88239100	-3.71136600
P	3.07673600	1.70723600	-1.69157800
P	3.29130000	-1.58230200	0.84208500
P	0.47539500	-0.63425300	3.75414900
P	2.57961100	2.38894500	1.80232400
P	-0.38655200	3.80848700	-0.77209000
Al	1.76558800	3.08713100	-0.30069300
Al	0.75029700	1.46280600	2.77562600
Al	-1.74776100	2.65798300	0.69535000
Al	-1.56355300	-0.11402500	2.76871500
Al	-0.76283800	-1.21479900	-2.74535300
Al	1.54289500	0.37285400	-2.70092900
Al	3.06616800	0.63839600	0.36177400
Al	-3.11660400	-0.35574200	-0.33261800
Al	-1.58667400	-2.60090400	0.33855300
Al	1.33546400	-1.87917100	2.05669300
Al	1.72812600	-2.42186700	-0.65483500
Al	-1.35831500	2.11503100	-2.00469200
O	2.76987100	4.77510700	-0.39560100
C	2.62288900	5.68117800	-1.51173100
H	3.45287500	6.38791500	-1.50102500
H	1.65691700	6.18647600	-1.45775900
H	2.67544200	5.05938600	-2.40581300
H	2.84197100	5.23791200	0.45637300

Complex A3

Absolute Energy = -7121.195808 au.

Cartesian atomic coordinates:

P	-2.04569300	-2.29810300	-1.64247500
P	1.88851500	-1.75713900	-2.93050900
P	1.13240400	-3.32804600	0.85939100
P	-2.51042000	-1.50758300	1.77496500
P	-1.25517100	2.35307000	2.75153100
P	-3.19149100	1.64222200	-0.87952500
P	-0.35931000	0.82864600	-3.83238700
P	3.12200300	2.10332500	-1.94278400
P	3.82663800	-1.03943700	0.70270600
P	0.99088700	-0.22795500	3.65224500
P	2.68238200	2.90899200	1.47657500
P	-0.49994700	3.93189600	-1.03568600
Al	1.58651700	3.16732300	-0.55595900
Al	1.00745800	1.84017800	2.58836500
Al	-1.67028900	2.68259000	0.53962700
Al	-1.12161600	0.03085600	2.71380400
Al	-0.37500200	-1.23915500	-2.76483700
Al	1.75488800	0.56161700	-2.90920800
Al	3.37819800	1.12358500	0.15757900
Al	-2.73081000	-0.51348300	-0.31606300
Al	-0.95569700	-2.55288300	0.39642100
Al	1.93168200	-1.45677300	1.98519000
Al	2.30178700	-2.07787300	-0.71511300
Al	-1.29553300	2.05731600	-2.16059800
O	6.49378800	2.91233100	-1.40386100
C	6.52895200	1.83848100	-0.49743400
H	6.28164600	0.87728700	-0.96955700
H	5.86095100	2.00480700	0.36843200
H	7.54595400	1.77804200	-0.10476100
H	5.63347200	2.91724100	-1.85338600

Complex A4

Absolute Energy = -7121.195668 au.

Cartesian atomic coordinates:

P	-2.39754500	-2.14102400	-1.65140300
P	1.57697900	-2.02158700	-2.92396300
P	0.63863700	-3.55077600	0.84189800
P	-2.78341600	-1.35022200	1.77549200
P	-1.11067100	2.33146000	2.81040800
P	-3.09909700	1.89236200	-0.83389000
P	-0.36736900	0.80879500	-3.79452300
P	3.23179200	1.66083300	-1.88003400
P	3.57226900	-1.57755100	0.72260000
P	0.83226100	-0.49555400	3.67996700
P	2.86901600	2.45927200	1.55119100
P	-0.16748600	3.86800000	-0.95299100
Al	1.81923900	2.86893100	-0.48136500
Al	1.08093000	1.57226800	2.64666400
Al	-1.47535500	2.73505100	0.60180000
Al	-1.23506800	0.00890400	2.74045600
Al	-0.61563800	-1.25921700	-2.75563400
Al	1.70077600	0.29684000	-2.86991900
Al	3.36737300	0.62713700	0.20554800
Al	-2.88303000	-0.30940000	-0.30206900
Al	-1.34952000	-2.54330800	0.38592300
Al	1.63793600	-1.79603600	1.99549500
Al	1.94535000	-2.41738600	-0.71281600
Al	-1.16540100	2.11077300	-2.10689900
O	6.72716600	1.78738400	-1.61178500
C	6.48638200	1.87093800	-0.22935700
H	6.03868700	0.94220500	0.17139600
H	5.84565200	2.72269600	0.03777800
H	7.45340900	1.99591100	0.26169300
H	5.87585500	1.79250800	-2.07778100

Complex A5

Absolute Energy = -7121.192348 au.

Cartesian atomic coordinates:

P	-2.81877000	-1.57718000	-1.71430500
P	1.05191100	-2.71448700	-2.78491200
P	-0.47064400	-3.76019900	0.95862100
P	-3.08957900	-0.58882300	1.67242900
P	-0.41281100	2.44068600	2.71230900
P	-2.28967000	2.50317700	-1.01484200
P	0.10692000	0.53867300	-3.80969400
P	3.72385000	0.31777700	-1.74206000
P	2.92814700	-2.77590000	0.94294500
P	0.53054100	-0.81210900	3.73849400
P	3.45857700	1.30363800	1.64661100
P	1.10915000	3.49016400	-1.03048400
Al	2.67356700	1.94548700	-0.44861900
Al	1.44562700	1.04323900	2.67517200
Al	-0.54398100	2.86142300	0.47985500
Al	-1.24064500	0.26695400	2.68461100
Al	-0.80739800	-1.31634400	-2.74443900
Al	1.87806700	-0.54030700	-2.75864700
Al	3.41247900	-0.63205000	0.35781900
Al	-2.77723500	0.36049700	-0.42820600
Al	-2.03305700	-2.20996000	0.38171200
Al	0.96892600	-2.35333800	2.12390500
Al	1.18250100	-3.13330500	-0.55261400
Al	-0.32962300	2.08028200	-2.19412100
O	6.02047500	4.86650700	-1.72270900
C	5.35987300	3.62116100	-1.68655400
H	5.05677300	3.35541800	-0.65935400
H	5.97156000	2.80353500	-2.08821200
H	4.46715300	3.72104000	-2.30951200
H	6.83654900	4.80859700	-1.20823300

Complex B1

Absolute Energy = -7160.534146 au.

Cartesian atomic coordinates:

P	1.19404000	14.83521300	0.06768200
P	3.68895100	13.09235300	-2.78674300
P	-0.35897200	13.71161800	-3.63878700
P	-2.02806800	13.36863500	0.17035600
P	-1.92309700	9.23154000	0.73481300
P	0.76223100	11.69753000	2.78199500
P	4.27274900	12.05347300	0.53915900
P	3.79783700	8.95970200	-2.24202300
P	0.99594100	10.65096500	-4.80221700
P	-2.50162100	10.27601600	-2.58961600
P	0.55854800	7.47778700	-2.15016400
P	2.10465400	8.66379000	1.55977100
Al	2.30921200	8.01280000	-0.65770900
Al	-1.14256300	8.78662800	-1.41605900
Al	0.07665800	9.75910000	1.68441700
Al	-2.22888800	11.19323100	-0.47284800
Al	2.93202600	13.54395200	-0.63704300
Al	3.97884700	11.12043500	-1.57608700
Al	1.72293300	9.14784400	-3.24590600
Al	0.02778800	13.20185300	1.24615000
Al	-0.43061700	14.02677000	-1.38767700
Al	-0.85935200	11.43709900	-3.65014900
Al	1.68093200	12.59299300	-3.73141100
Al	2.61022300	10.91625300	1.59843900
O	3.10732200	6.27472900	-0.28022200
C	3.77234100	5.41159600	-1.24193500
C	5.27613800	5.52897900	-1.12727200
H	3.40853200	4.39602100	-1.06850000
H	3.40681200	5.75469700	-2.21242400
H	5.75130700	4.88751600	-1.87523900
H	5.59160500	6.56115900	-1.30190400
H	5.62579900	5.20679700	-0.14028800
H	3.41287500	6.14091200	0.63378200

Complex B2

**Absolute Energy = -7160.533259 au.**			
**Cartesian atomic coordinates:**			
P	2.92444100	14.87613000	1.68234300
P	5.26839800	13.78419500	-1.59180000
P	1.20979100	14.65651300	-2.11445100
P	-0.32965000	13.48155500	1.58277000
P	-0.31836900	9.32368600	1.18922300
P	2.50601900	11.21172000	3.63163700
P	5.94493200	12.00699700	1.38541500
P	5.30385900	9.63896500	-2.01600000
P	2.44170200	11.91635700	-3.99455500
P	-0.98570500	11.11058200	-1.78688700
P	2.02680400	8.24140800	-2.10005200
P	3.72146700	8.51750800	1.69438600
Al	3.87379300	8.39074000	-0.61158200
Al	0.37443100	9.37198300	-1.03623300
Al	1.73158600	9.59887600	2.13924800
Al	-0.61611800	11.51530900	0.47086400
Al	4.60129900	13.74756100	0.63233000
Al	5.55559200	11.58328700	-0.87338300
Al	3.19266400	10.09126900	-2.84714400
Al	1.75979200	13.03895900	2.50683100
Al	1.22695500	14.45015000	0.15023200
Al	0.64943300	12.45392900	-2.62194600
Al	3.21532600	13.55174300	-2.54141100
Al	4.29198900	10.68934500	2.22751800
O	4.65838600	6.59847400	-0.81197600
C	4.33484200	5.45795100	0.04013100
C	2.87521300	5.07162800	-0.06671800
H	5.00813300	4.64976700	-0.25514100
H	4.59791200	5.79088700	1.04480500
H	2.69180800	4.18715800	0.55117900
H	2.22949800	5.87978100	0.29055800
H	2.59575000	4.83646400	-1.09859300
H	4.69815800	6.33416600	-1.74775100

Complex B3

Absolute Energy = -7160.533767 au.			
**Cartesian atomic coordinates:**			
P	3.06566800	15.30636700	1.05916400
P	5.42748900	13.32397000	-1.75130300
P	1.46310900	14.29658400	-2.65937800
P	-0.27610700	14.13617000	1.13011900
P	-0.55568800	10.01361400	1.72755100
P	2.31899700	12.24318400	3.79056400
P	5.87234100	12.25819600	1.58771300
P	5.17415000	9.20173300	-1.19293100
P	2.55016600	11.11617400	-3.78231200
P	-0.99264100	11.07507500	-1.61347200
P	1.80891000	8.02615200	-1.09803000
P	3.39349800	9.08946400	2.60558700
Al	3.60825300	8.42540200	0.39369600
Al	0.21311000	9.48304300	-0.40780000
Al	1.47457600	10.37080100	2.69122900
Al	-0.66859400	11.98362300	0.49968400
Al	4.68655400	13.85522000	0.38529600
Al	5.52838900	11.34110100	-0.52705400
Al	3.12733800	9.56772800	-2.20716300
Al	1.74399500	13.79418600	2.23304300
Al	1.39224900	14.63689400	-0.41170600
Al	0.75967700	12.07695800	-2.66130700
Al	3.39499100	12.99761300	-2.71735800
Al	4.10619900	11.28430000	2.64290200
O	4.26764800	6.62854400	0.78008300
C	4.33738600	5.55621300	-0.19967100
C	5.34805500	4.51905800	0.23671700
H	3.33145900	5.14862600	-0.33101200
H	4.63914900	6.05643100	-1.12254600
H	5.41849800	3.73569700	-0.52322300
H	6.33426200	4.97231100	0.36362800
H	5.05130300	4.04314100	1.17763100
H	4.05954800	6.30820100	1.67553400

Complex B4

**Absolute Energy = -7160.532923 au.**			
**Cartesian atomic coordinates:**			
P	1.31857300	14.65582600	0.39851300
P	3.51897200	13.29272400	-2.87559700
P	-0.58353900	14.01250800	-3.25990600
P	-1.89552100	13.18851700	0.61606100
P	-1.78990300	9.01274300	0.63429300
P	1.10472100	11.19193200	2.71607300
P	4.39915000	11.84325900	0.22256800
P	3.63862300	9.11738500	-2.86193600
P	0.62476600	11.12221000	-4.91692100
P	-2.66953200	10.48099200	-2.45973800
P	0.37137600	7.64449100	-2.68201900
P	2.29668300	8.34973500	0.97721500
Al	2.27121700	7.97321100	-1.31129100
Al	-1.23356800	8.84831400	-1.62193700
Al	0.30233700	9.41572600	1.43742100
Al	-2.18435900	11.11487400	-0.27753000
Al	2.97123000	13.46906000	-0.62496900
Al	3.89114000	11.18143800	-1.95454900
Al	1.48905900	9.42862000	-3.65213800
Al	0.25057200	12.88340300	1.46241400
Al	-0.44306600	14.03971700	-0.98990500
Al	-1.10877600	11.75822600	-3.51117900
Al	1.42703900	12.91556500	-3.68101600
Al	2.82413800	10.57827800	1.26840900
O	2.91536100	6.13449300	-1.41531500
C	3.97160500	5.53170000	-0.62132300
C	3.39695500	4.65538700	0.46958800
H	4.62241200	4.98679500	-1.30938500
H	4.52181000	6.38178200	-0.21222000
H	4.21070800	4.22778800	1.06266500
H	2.75449400	5.24342000	1.13061300
H	2.81841800	3.82582800	0.04851600
H	2.29212500	5.48814300	-1.78940200

Complex B5

Absolute Energy = -7160.532602 au.			
**Cartesian atomic coordinates:**			
P	2.95515400	15.08701600	1.51823700
P	5.29763000	13.78599900	-1.67815500
P	1.25721900	14.67573600	-2.27276500
P	-0.31935000	13.74413100	1.47855100
P	-0.37783700	9.57192000	1.32045500
P	2.47240100	11.53976500	3.66094200
P	5.92884500	12.15684200	1.39507800
P	5.27523900	9.62140800	-1.87058700
P	2.45389900	11.80951000	-3.98018500
P	-1.00256800	11.19685500	-1.75439400
P	1.95230700	8.24908800	-1.89304800
P	3.64634200	8.72009600	1.86878800
Al	3.79289100	8.47514000	-0.43290000
Al	0.32790400	9.48313500	-0.89897700
Al	1.67535600	9.85546800	2.26092900
Al	-0.63492900	11.72292000	0.47854200
Al	4.61910800	13.87791300	0.54255600
Al	5.54515100	11.62559000	-0.84014000
Al	3.16647300	10.02509300	-2.74615300
Al	1.75793800	13.31490400	2.43598600
Al	1.25968500	14.60151000	0.00103600
Al	0.66279700	12.45674900	-2.65331400
Al	3.24654300	13.51737500	-2.62213400
Al	4.24848000	10.90789000	2.29044800
O	4.61434600	6.70632300	-0.59706700
C	4.12610600	5.54935700	0.13633700
C	4.62915700	4.27616400	-0.50668100
H	4.43079200	5.65342800	1.18138000
H	3.03875400	5.63508100	0.07439100
H	4.22618300	3.41238200	0.02953600
H	4.31092000	4.21937800	-1.55052000
H	5.72190400	4.21270700	-0.46340400
H	5.58378000	6.71243200	-0.68169700

Complex B6

**Absolute Energy = -7160.495437 au.**			
**Cartesian atomic coordinates:**			
P	0.98174900	14.73720200	0.24292400
P	3.36160000	13.68654300	-3.02073100
P	-0.78986000	13.80358800	-3.41965200
P	-1.99701400	12.84041000	0.45874000
P	-1.31857300	8.72232000	0.51006700
P	1.24900400	11.28558600	2.57422300
P	4.42152600	12.37749000	0.09515500
P	4.02847900	9.57329000	-2.96495700
P	0.79546400	11.11556500	-5.08649100
P	-2.37739500	10.02880200	-2.60339400
P	1.05484300	7.65766000	-2.75595500
P	2.83402700	8.59631100	0.90756600
Al	2.71365000	8.50425600	-1.36189600
Al	-0.74193400	8.61968200	-1.73999700
Al	0.69316800	9.40470800	1.32442900
Al	-1.99436400	10.74349300	-0.42308300
Al	2.78261400	13.78576900	-0.76885400
Al	4.04924800	11.67374300	-2.08697400
Al	1.86769400	9.55594700	-3.82742700
Al	0.16780900	12.83766300	1.31047800
Al	-0.66967300	13.87219100	-1.14854500
Al	-1.00577400	11.50085900	-3.66520200
Al	1.35160500	12.99659900	-3.83513300
Al	3.04820900	10.90048000	1.15075200
O	5.82359100	6.61049800	-3.87514300
C	4.61954700	5.98799200	-3.47240400
C	4.56405100	5.75600400	-1.96743300
H	3.74866100	6.57185200	-3.80748300
H	4.58313900	5.03000700	-3.99853200
H	3.60557500	5.31667400	-1.66656800
H	5.37037400	5.08688600	-1.65327700
H	4.71327000	6.69762500	-1.41905400
H	5.78124000	7.54790500	-3.62677000

Complex B7

**Absolute Energy = -7160.49578 au.**			
**Cartesian atomic coordinates:**			
P	1.74874700	14.50863800	0.47601800
P	3.90297700	12.70564900	-2.60681300
P	0.00099500	14.01330800	-3.28383500
P	-1.66348200	13.57245200	0.51742900
P	-2.23328200	9.44088700	0.67125000
P	0.85036700	11.20931100	2.86751200
P	4.34652600	11.23786600	0.58810300
P	3.32407900	8.58312300	-2.46184400
P	0.82303000	10.92916100	-4.80818400
P	-2.66908300	10.89484200	-2.52439400
P	-0.07954000	7.62127200	-2.41099400
P	1.66503600	8.12258000	1.34627500
Al	1.66675400	8.01067100	-0.92589500
Al	-1.57440900	9.09823900	-1.53258900
Al	-0.16318800	9.53642800	1.60922200
Al	-2.22630800	11.53891600	-0.33342400
Al	3.24750800	13.03848600	-0.39835800
Al	3.91009100	10.61062400	-1.60611600
Al	1.30633600	9.16103700	-3.46394600
Al	0.34917300	12.96754000	1.51393300
Al	0.00291800	14.11677800	-1.01105700
Al	-0.86620100	11.86754900	-3.51290700
Al	1.83185900	12.60365100	-3.54541800
Al	2.53787100	10.26631700	1.57318200
O	5.17394100	5.71378900	-1.54373100
C	4.58217100	5.76319300	-0.26234600
C	5.14254900	6.88083700	0.60530100
H	3.48337200	5.85140900	-0.34161500
H	4.78222300	4.78991900	0.19425700
H	4.70353800	6.85445000	1.60857300
H	6.22856000	6.77935800	0.68930000
H	4.92744000	7.86556900	0.16950800
H	4.93387100	6.52048400	-2.02830200

Complex B8

Absolute Energy = -7160.492422 au.

Cartesian atomic coordinates:

P	2.17423000	2.65349400	-10.46341300
P	5.75024900	3.30852500	-8.41173900
P	3.97256100	-0.46861100	-8.35750700
P	-0.06208600	0.53930000	-8.71642800
P	-0.90058700	1.87766000	-4.85314700
P	-0.54523300	4.61955700	-7.98079900
P	3.40464800	5.94965900	-8.21676400
P	4.91595100	4.65197500	-4.54722500
P	5.39417700	0.56697200	-5.28584800
P	1.44587000	-0.76366600	-5.04864900
P	2.67618400	2.53371800	-2.80382400
P	0.87724600	5.65320800	-4.90969100
Al	2.64232000	4.46714200	-4.09966100
Al	1.19541600	1.30811700	-4.02115100
Al	-0.33829800	3.87505800	-5.78672000
Al	0.07114300	0.48910600	-6.44602700
Al	3.65478200	3.87879100	-9.24481500
Al	4.77690100	4.69590800	-6.81720100
Al	4.50504900	2.38313100	-4.23973200
Al	0.35825100	2.80766300	-9.01793600
Al	2.21172600	0.71989100	-9.17159000
Al	3.42198500	-0.35745200	-6.10063000
Al	5.18783900	1.31101500	-7.48022300
Al	1.42751000	5.54590100	-7.16675100
O	6.96783600	4.57209500	-0.75521200
C	6.00563300	3.53901200	-0.86828500
C	6.54320600	2.33090100	-1.62268500
H	5.63526100	3.23696200	0.12139800
H	5.16410000	3.98736400	-1.40576000
H	5.77342500	1.55523600	-1.72448200
H	6.90630400	2.63098900	-2.61349300
H	7.38732800	1.87512200	-1.09174600
H	7.71541700	4.25003700	-0.23198700

Complex B9

**Absolute Energy = -7160.491735 au.**			
**Cartesian atomic coordinates:**			
P	0.93729500	14.86920500	0.54010700
P	3.32783100	13.80254000	-2.71195100
P	-0.79595300	14.22636300	-3.20293600
P	-2.16796900	13.17236300	0.59528300
P	-1.76630100	9.01866300	0.46858800
P	0.90618000	11.30688900	2.71469800
P	4.21169800	12.28684500	0.36049800
P	3.72919000	9.64909100	-2.83843100
P	0.65455900	11.51463900	-4.95491500
P	-2.65085500	10.53468600	-2.60331400
P	0.62090800	7.94969900	-2.78663800
P	2.35620400	8.59756100	0.95908700
Al	2.30136800	8.61116400	-1.31827500
Al	-1.13459400	8.97962100	-1.76949400
Al	0.26244000	9.52518600	1.36434700
Al	-2.27920200	11.12134800	-0.38349400
Al	2.69608700	13.84238000	-0.47367100
Al	3.83878800	11.69971000	-1.85894800
Al	1.59153100	9.83861500	-3.73446700
Al	-0.03325200	12.98433500	1.49906100
Al	-0.73006300	14.18242500	-0.92918300
Al	-1.15494700	11.95570200	-3.56096800
Al	1.29794800	13.29670000	-3.60610400
Al	2.71244200	10.86782300	1.31660600
O	4.63163600	3.83085300	-0.31965100
C	4.61513000	4.43600700	-1.59948000
C	3.88462300	5.77408600	-1.59728700
H	5.63612700	4.56150700	-1.98574700
H	4.09874300	3.72581100	-2.24968200
H	3.83076600	6.18574900	-2.61207100
H	2.86913900	5.63658000	-1.20847900
H	4.41413100	6.49624100	-0.96114400
H	5.10874900	4.40601900	0.29495800

Complex B10

**Absolute Energy = -7160.491729 au.**			
**Cartesian atomic coordinates:**			
P	1.09273800	15.04771700	0.12518800
P	3.57885200	13.40045400	-2.79482400
P	-0.47005600	14.01680000	-3.60456900
P	-2.12523800	13.57733400	0.20283900
P	-2.01886100	9.42785800	0.64872100
P	0.68437400	11.82317300	2.74310700
P	4.17754800	12.26007200	0.50084200
P	3.68754300	9.25141900	-2.35141000
P	0.87545100	11.00524300	-4.88665400
P	-2.61728700	10.56721700	-2.64703600
P	0.46682400	7.77904100	-2.27170200
P	2.02853800	8.81277300	1.45791000
Al	2.11050500	8.52560700	-0.79968800
Al	-1.26571100	9.04720600	-1.51859400
Al	-0.01049900	9.92203700	1.59730400
Al	-2.33089500	11.42453100	-0.50351500
Al	2.82685700	13.78119900	-0.62666000
Al	3.89133400	11.40380600	-1.64252100
Al	1.62109600	9.45517700	-3.39769100
Al	-0.06263900	13.37750000	1.25997200
Al	-0.53554800	14.27604000	-1.34521900
Al	-0.97160600	11.74713300	-3.68360800
Al	1.57065400	12.90869600	-3.74431500
Al	2.53055700	11.08176000	1.53734900
O	5.46133300	4.19974200	-0.95094900
C	4.12230100	4.19663100	-0.49092500
C	3.46316100	5.56499000	-0.62291600
H	3.52944900	3.43712200	-1.01917100
H	4.17709800	3.90385500	0.56027900
H	2.44983700	5.54221500	-0.20322800
H	4.06095300	6.31298500	-0.08953400
H	3.39139300	5.85751100	-1.67860500
H	5.47017800	4.41992400	-1.89306400
